# Outbreaks of Illness Associated with Recreational Water — United States, 2011–2012

**Published:** 2015-06-26

**Authors:** Michele C. Hlavsa, Virginia A. Roberts, Amy M. Kahler, Elizabeth D. Hilborn, Taryn R. Mecher, Michael J. Beach, Timothy J. Wade, Jonathan S. Yoder

**Affiliations:** 1 Division of Foodborne, Waterborne, and Environmental Diseases, National Center for Emerging and Zoonotic Infectious Diseases, CDC; 2 Environmental Protection Agency; 3 Oak Ridge Institute for Science and Education, Oak Ridge, Tennessee

Outbreaks of illness associated with recreational water use result from exposure to chemicals or infectious pathogens in recreational water venues that are treated (e.g., pools and hot tubs or spas) or untreated (e.g., lakes and oceans). For 2011–2012, the most recent years for which finalized data were available, public health officials from 32 states and Puerto Rico reported 90 recreational water–associated outbreaks to CDC’s Waterborne Disease and Outbreak Surveillance System (WBDOSS) via the National Outbreak Reporting System (NORS). The 90 outbreaks resulted in at least 1,788 cases, 95 hospitalizations, and one death. Among 69 (77%) outbreaks associated with treated recreational water, 36 (52%) were caused by *Cryptosporidium*. Among 21 (23%) outbreaks associated with untreated recreational water, seven (33%) were caused by *Escherichia coli* (*E. coli* O157:H7 or *E. coli* O111). Guidance, such as the Model Aquatic Health Code (MAHC), for preventing and controlling recreational water–associated outbreaks can be optimized when informed by national outbreak and laboratory (e.g., molecular typing of *Cryptosporidium*) data.

A recreational water–associated outbreak is the occurrence of similar illnesses in two or more persons, epidemiologically linked by location and time of exposure to recreational water or recreational water–associated chemicals volatilized into the air surrounding the water. Public health officials in the 50 states, the District of Columbia, U.S. territories, and Freely Associated States* voluntarily report outbreaks of recreational water–associated illness to CDC. In 2010, waterborne outbreaks became nationally notifiable. This report summarizes data on recreational water–associated outbreaks electronically reported by October 30, 2014 to CDC’s WBDOSS (http://www.cdc.gov/healthywater/surveillance/) for 2011 and 2012 via NORS.† Data requested for each outbreak include the number of cases,§ hospitalizations, and deaths; etiology; setting (e.g., hotel) and venue (e.g., hot tub or spa) where the exposure occurred; earliest illness onset date; and illness type. All outbreaks are classified according to the strength of data implicating recreational water as the outbreak vehicle (1).¶ Outbreak reports classified as Class I have the strongest supporting epidemiologic, clinical laboratory and environmental health data, and those classified as Class IV, the weakest. Classification does not assess adequacy or completeness of investigations.** Negative binomial regression (PROC GENMOD in SAS 9.3 [Cary, NC]) was used to assess trends in the number of outbreaks over time.

For the years 2011 and 2012, public health officials from 32 states and Puerto Rico reported 90 recreational water–associated outbreaks (http://www.cdc.gov/healthywater/surveillance/rec-water-tables-figures.html) (Figure 1), which resulted in at least 1,788 cases, 95 (5%) hospitalizations, and one death. Etiology was confirmed for 73 (81%) outbreaks: 69 (77%) outbreaks were caused by infectious pathogens, including two outbreaks with multiple etiologies, and four (4%) by chemicals (Table). Among the outbreaks caused by infectious pathogens, 37 (54%) were caused by *Cryptosporidium*. On the basis of data reported to CDC, 37 (41%) of the 90 outbreak reports were categorized as class IV.

Outbreaks associated with treated recreational water accounted for 69 (77%) of the 90 outbreaks reported for 2011–2012, and resulted in at least 1,309 cases, 73 hospitalizations, and one reported death. The median number of cases reported for these outbreaks was seven (range: 2–144 cases). Hotels (e.g., hotel, motel, lodge, or inn) were the setting of 13 (19%) of the treated recreational water–associated outbreaks. Twelve (92%) of these 13 outbreaks started outside of June–August; ten (77%) were at least in part associated with a spa. Among the 69 outbreaks, 36 (52%) were caused by *Cryptosporidium*. The 69 outbreaks had a seasonal distribution, with 42 (61%) starting in June–August (Figure 1). Acute gastrointestinal illness was the disease manifestation in 34 (81%) of these summer outbreaks, with *Cryptosporidium* causing 32 (94%) of them. Since 1988, the year that the first U.S. treated recreational water–associated outbreak of cryptosporidiosis was detected (2,3) (Figure 2), the number of these outbreaks reported annually (range: 0–40 outbreaks) has significantly increased (negative binomial regression; p<0.001). Incidence of these cryptosporidiosis outbreaks has also, at least in part, driven the significant increase (negative binomial regression; p<0.001) in the overall number of recreational water–associated outbreaks reported annually (range: 6–84).

**Summary**

What is already known on this topic?

Treated and untreated recreational water–associated outbreaks occur throughout the United States and their incidence has been increasing in recent years. CDC collects data on waterborne outbreaks electronically submitted by the 50 states, the District of Columbia, U.S. territories, and Freely Associated States to CDC’s Waterborne Disease and Outbreak Surveillance System via the National Outbreak Reporting System.

What is added by this report?

For 2011–2012, a total of 90 recreational water–associated outbreaks were reported to CDC, resulting in at least 1,788 cases, 95 hospitalizations, and one death. *Cryptosporidium* caused over half of the outbreaks associated with treated recreational water venues (e.g., pools). *Escherichia coli* O157:H7 and O111 caused one third of outbreaks associated with untreated recreational water (e.g., lakes).

What are the implications for public health practice?

Guidance, such as the Model Aquatic Health Code (MAHC), to prevent and control recreational water–associated outbreaks can be optimized when informed by national outbreak and laboratory (e.g., molecular typing of *Cryptosporidium*) data.

For 2011–2012, 21 (23%) outbreaks were associated with untreated recreational water. These outbreaks resulted in at least 479 cases and 22 hospitalizations. The median number of cases reported for these outbreaks was 16 (range: 2–125). Twenty (95%) of these outbreaks were associated with fresh water; 18 (86%) began in June–August; and seven (33%) were caused by *E. coli* O157:H7 or O111. One outbreak associated with exposure to cyanobacterial toxins was reported.

## Discussion

*Cryptosporidium* continues to be the dominant etiology of recreational water–associated outbreaks. Half of all treated recreational water–associated outbreaks reported for 2011–2012 were caused by *Cryptosporidium*. Among treated recreational water–associated outbreaks of gastrointestinal illness that began in June–August, >90% were caused by *Cryptosporidium,* an extremely chlorine-tolerant parasite that can survive in water at CDC-recommended chlorine levels (1–3 mg/L) and pH (7.2–7.8) for >10 days (4). In contrast, among 14 untreated recreational water–associated outbreaks of gastrointestinal illness starting in June–August, 7% (one) were caused by *Cryptosporidium*. The decreased diversity of infectious etiologies causing treated recreational water–associated outbreaks is likely a consequence of the aquatic sector’s reliance on halogen disinfection (e.g., chlorine or bromine) and maintenance of proper pH, which are well documented to inactivate most infectious pathogens within minutes (5). Continued reporting of treated recreational water–associated outbreaks caused by chlorine-intolerant pathogens (e.g., *E. coli* O157:H7 and norovirus) highlights the need for continued vigilance in maintaining water quality (i.e., disinfectant level and pH), as has been recommended for decades (5).

In the United States, codes regulating public treated recreational water venues are independently written and enforced by individual state or local agencies; the consequent variation in the codes is a potential barrier to preventing and controlling outbreaks associated with these venues. In August 2014, CDC released the first edition of MAHC (http://www.cdc.gov/mahc), a comprehensive set of science-based and best-practice recommendations to reduce risk for illness and injury at public, treated recreational water venues. MAHC represents the culmination of a 7-year, multi-stakeholder effort and is an evolving resource that addresses emerging public health threats, such as treated recreational water-associated outbreaks of cryptosporidiosis, by incorporating the latest scientifically validated technologies that inactivate or remove infectious pathogens. For example, MAHC recommends additional water treatment (e.g., ultraviolet light or ozone) to inactivate *Cryptosporidium* oocysts at venues where WBDOSS data indicate there is increased risk for transmission. MAHC recommendations can be voluntarily adopted, in part or as a whole, by state and local jurisdictions.

The number of reported untreated recreational water–associated outbreaks confirmed or suspected to be caused by cyanobacterial toxins has decreased, from 11 (2009–2010) to one (2011–2012) (6). This decrease is likely the result of a decrease in outbreak reporting rather than a true decrease in incidence. CDC is currently developing a mechanism for reporting algal bloom–associated individual cases through NORS to better characterize their epidemiology.

The findings in this report are subject to at least two limitations. First, the outbreak counts presented are likely an underestimate of actual incidence. Many factors can present barriers to the detection, investigation, and reporting of outbreaks: 1) mild illness; 2) small outbreak size; 3) long incubation periods; 4) wide geographic dispersion of ill swimmers; 5) transient nature of contamination; 6) setting or venue of outbreak exposure (e.g., residential backyard pool); and 7) potential lack of communication between those who respond to outbreaks of chemical etiology (e.g., hazardous materials personnel) and those who usually report outbreaks (e.g., infectious disease epidemiologists). Second, because of variation in public health capacity and reporting requirements across jurisdictions, those reporting outbreaks most frequently might not be those in which outbreaks most frequently occur.

Increasingly, molecular typing tools are being employed to understand the epidemiology of waterborne disease and outbreaks. Most species and genotypes of *Cryptosporidium* are morphologically indistinguishable from one another, and only molecular methods can distinguish species and subtypes and thereby elucidate transmission pathways (7,8). Systematic national genotyping and subtyping of *Cryptosporidium* in clinical specimens and environmental samples through CryptoNet (http://www.cdc.gov/parasites/crypto/cryptonet.html) can identify circulating *Cryptosporidium* species and subtypes and help identify epidemiologic linkages between reported cases. Molecular typing could substantially help elucidate cryptosporidiosis epidemiology in the United States and inform development of future guidance to prevent recreational water–associated and other outbreaks of cryptosporidiosis (9,10).

## Figures and Tables

**FIGURE 1:**
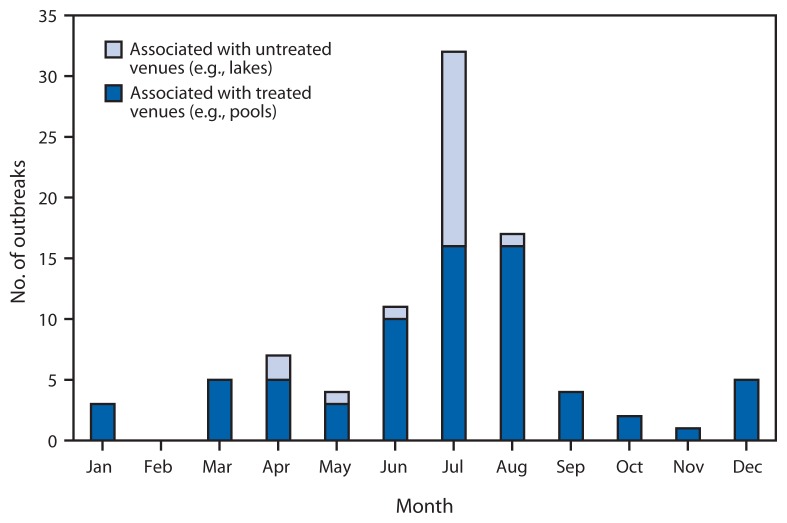
Number* of outbreaks associated with recreational water, by month — United States, 2011–2012^†^ * Total n = 90. ^†^ Numbers for 2011 and 2012 are combined for each month.

**FIGURE 2:**
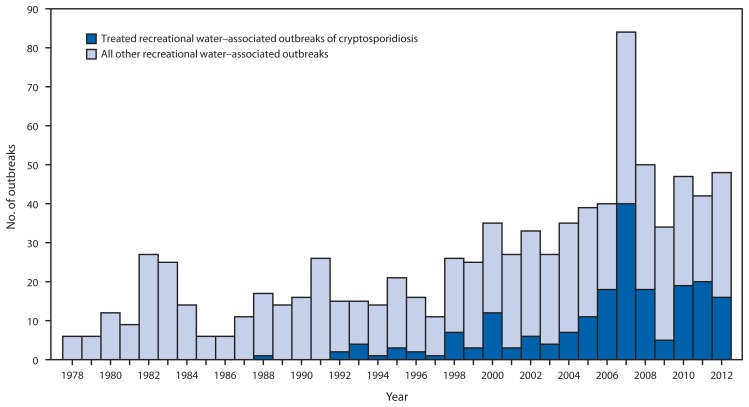
Number* of outbreaks associated with recreational water, by year — United States, 1978–2012 * Total n = 879.

**TABLE:** Number* of outbreaks, cases, and hospitalizations associated with recreational water, by etiology and type of water exposure — United States, 2011–2012

	Type of exposure						Total for treated and untreated exposure					
		
	Treated			Untreated			Outbreaks		Cases†		Hospitalized	
					
Etiology	Outbreaks	Cases†	Hospitalized	Outbreaks	Cases	Hospitalized	No.	(%)	No.	(%)	No.	(%)§
**Bacterium**	14	75	24	7	76	18	21	(23)	151	(8)	42	(44)
*Escherichia coli* O111	0	0	0	2	11	0	2		11		0	
*Escherichia coli* O157:H7	2	21	5	3	31	15	5		52		20	
*Legionella* spp.	9	33	18	0	0	0	9		33		18	
*Pseudomonas aeruginosa*	2	16	0	0	0	0	2		16		0	
*Shigella sonnei*	1	5	1	2	34	3	3		39		4	
**Parasite**	37	895	44	4	72	0	41	(46)	967	(54)	44	(46)
Avian schistosomes	0	0	0	1	43	0	1		43		0	
*Cryptosporidium* spp.	36	874	44	1	16	0	37		890		44	
*Giardia intestinalis*	1	21	0	2	13	0	3		34		0	
**Virus**	2	122	0	3	85	1	5	(6)	207	(12)	1	(1)
Adenovirus	0	0	0	1	32	1	1		32		1	
Norovirus	2	122	0	2	53	0	4		175		0	
**Chemical**	3	57	0	1	8	0	4	(4)	65	(4)	0	(0)
Chlorine	2	46	0	0	0	0	2		46		0	
Chlorine gas	1	11	0	0	0	0	1		11		0	
Cyanobacterial toxin(s)	0	0	0	1	8	0	1		8		0	
**Multiple** ¶	0	0	0	2	181	2	2	(2)	181	(10)	2	(2)
*Giardia intestinalis,* norovirus	0	0	0	1	125	1	1		125		1	
*Escherichia coli, Plesiomonas shigelloides, Shigella sonnei*	0	0	0	1	56	1	1		56		1	
**Unidentified**	13	160	5	4	57	1	17	(19)	217	(12)	6	6
Suspected avian schistosomes	0	0	0	3	22	1	3		22		1	
Suspected pool chemical	1	3	0	0	0	0	1		3		0	
Suspected chloramine	2	13	0	0	0	0	2		13		0	
Suspected chlorine	1	12	0	0	0	0	1		12		0	
Suspected chlorine gas	1	3	0	0	0	0	1		3		0	
Suspected *Legionella* spp.	2	52	1	0	0	0	2		52		1	
Suspected norovirus	2	21	4	1	35	0	3		56		4	
Suspected *P. aeruginosa*	4	56	0	0	0	0	4		56		0	
**Total**	**69**	**1,309**	**73**	**21**	**479**	**22**	**90**		**1,788**		**95**	
**(%)**	**(77)**	**(73)**	**(77)**	**(23)**	**(27)**	**(23)**		**(100)**		**(100)**		**(100)**

* n = 90.

† One death was reported for an outbreak-related case of legionellosis.

§ Percentages do not add up to 100% because of rounding.

¶ Defined as outbreaks in which more than one type of etiologic agent (e.g., bacterium or virus) is detected in specimens from affected persons. Clinical test results were historically reported to CDC at the clinical specimen level (e.g., five of 10 stool specimens tested positive for *Cryptosporidium*). Multiple etiologies were assigned when each etiologic agent was found in ≥5% of positive clinical specimens. However, clinical test results are reported at the person level (e.g., five of 10 persons tested positive for *Cryptosporidium*) in the National Outbreak Reporting System. Therefore previously published data on multiple etiology assignments might not be directly comparable to such data presented in this report.

## Footnotes

* Includes Marshall Islands, Federated States of Micronesia, and Republic of Palau.

† Forms and guidance available at http://www.cdc.gov/nors/forms.html ; outbreaks resulting from recreational water exposures on cruise ships are not reported to WBDOSS.

§ If based on the estimated number of total cases, reporting agencies were not asked to provide supporting evidence.

¶ Classes delineated at http://www.cdc.gov/healthywater/surveillance/recreational/outbreak-classifications.html.

** Outbreaks and subsequent investigations occur under different circumstances, and not all outbreaks can be vigorously investigated. Multiple factors contribute to the ability to collect and report optimal epidemiologic, clinical laboratory, and environmental health data.
